# Massachusetts

**Published:** 1895-06

**Authors:** 


### MASSACHUSETTS.

The Haverhill *Bulletin*, Haverhill, Mass., of May 14, 1895, strongly urges the Governor to sign the bill which appropriates but $100,000 to the State Cattle Commission, and in so doing holds the Commission up to severe criticism, and further suggests that they should resign, owing to many errors made and extravagant use of the funds already appropriated. It suggests the creation of a State Veterinarian in place of a Commission.

The Tuberculosis Commission Bill of Massachusetts has been declared unconstitutional by the State's Attorney on the ground that the paying of full value for diseased cattle is forbidden by State statutes. It is intimated that the present Commission would prefer its veto rather than its passage in its present shape.

